# Multifunctional exosome-mimetics for targeted anti-glioblastoma therapy by manipulating protein corona

**DOI:** 10.1186/s12951-021-01153-3

**Published:** 2021-12-06

**Authors:** Jun-Yong Wu, Yong-Jiang Li, Jiemin Wang, Xiong-Bin Hu, Si Huang, Shilin Luo, Da-Xiong Xiang

**Affiliations:** 1 Department of Pharmacy, The Second Xiangya Hospital of Central South University, 139 Middle Renmin Road, Changsha, 410011 China; 2 Hunan Provincial Engineering Research Centre of Translational Medicine and Innovative Drug, Changsha, China; 3 Institute of Clinical Pharmacy, Central South University, Changsha, China; 4 Regenerative Medicine Institute (REMEDI), School of Medicine, College of Medicine, Nursing and Health Sciences, National University of Ireland Galway, Galway, Ireland

**Keywords:** Blood–brain barrier, Exosomes, Exosome-mimetics, Drug delivery, Glioblastoma

## Abstract

Targeted drug delivery to the glioblastoma (GBM) overcoming blood–brain barrier (BBB) has been challenging. Exosomes are promising vehicles for brain tumor drug delivery, but the production and purification hinder its application for nanomedicine. Besides, the formation of protein corona (PC) may affect the behaviour of nanocarriers. Here, multifunctional exosomes-mimetics (EM) are developed and decorated with angiopep-2 (Ang) for enhancing GBM drug delivery by manipulating PC. Docetaxel (DTX)-loaded EM with Ang modification (DTX@Ang-EM) show less absorption of serum proteins and phagocytosis by macrophages. Ang-EM show enhanced BBB penetration ability and targeting ability to the GBM. Ang-EM-mediated delivery increase the concentration of DTX in the tumor area. The multifunctional DTX@Ang-EM exhibits significant inhibition effects on orthotopic GBM growth with reduced side effects of the chemotherapeutic. Findings from this study indicate that the developed DTX@Ang-EM provide a new strategy for targeted brain drug delivery and GBM therapy.

**Supplementary Information:**

The online version contains supplementary material available at 10.1186/s12951-021-01153-3.

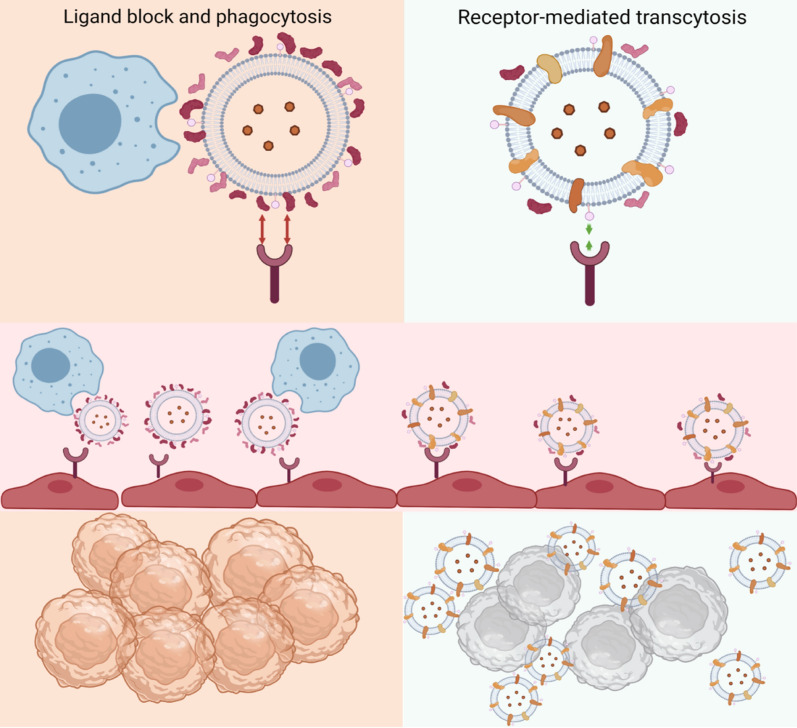

## Introduction

Glioblastoma (GBM) is the most-aggressive malignant tumor in the brain with five-year survival rate of about 5% [1]. GBM is invasive with poor prognosis and high mortality. Chemotherapy has been a mainstay in treating GBM [2], but the delivery of therapeutics to GBM is greatly impeded by the blood–brain barrier (BBB), which is composed mainly of brain endothelial cells, pericytes, and astrocytes [3], leading to limited treatment efficacy and serious side effects.

In recent years, nanomedicine-based strategies are emerging as promising approaches for improving brain tumor drug delivery [4, 5] and immunotherapy [6]. Various delivery systems have been developed to improve the therapeutic index while reducing side effects of drugs for GBM treatment. It has been reported that exosomes, endogenous nano-sized vesicles, released by almost all living cells, may have abilities to cross biological barriers such as BBB, offering efficient brain drug delivery [7–10]. The production and purification of exosomes are major challenges for their therapeutic applications [11], depite efforts for improvements [12]. Therefore, the development of exosome-mimetics (EM) as bioinspired delivery systems is of great interests for advanced drug delivery [13, 14]. Owing to the nanosize and lipid bilayer membranes of exosomes, liposomes hold great potentials for mimicking exosomal structure. Exosomes are lipid bilayer vesicles, which are similar to the structure of classical liposomes. Besides, the size distribution of liposomes is controllable and could be manipulated to approach that of exosomes. Previous studies have developed biomimetic liposomes for drug delivery [15, 16]. However, biomimetic liposomes may still have limited ability for crossing the BBB. In recent years, several ligands such as transferrin [17–19], apolipoprotein E [20–22] and cyclic internalizing peptide [23–25] have been reported to be able to facilitate the penetration of nanoparticle to BBB for brain drug delivery. Of note, angiopep-2 (Ang) is a promising ligand bind specifically to lipoprotein receptor-related protein 1 (LRP1) receptor and can improve the BBB transport efficiency of nanoparticles for brain drug delivery [26–28].

Nanoparticles interact with components in circulation after injection, leading to the formation of “protein corona” (PC), which changes the properties of nanoparticles and impact their in vivo fate [29]. It has been reported that the formation of PC on nanoparticles may hinder significantly the interaction between targeting ligand and receptor, thus losing targeting capabilities of ligand such as bicyclononyne [30] and transferrin [31]. Moreover, the PC on synthetic nanoparticle would impact the receptor targeting, lysosomal escape, and BBB transcytosis [32]. However, there are also studies reporting that targeting antibodies on nanoparticles could retain their targeting capacity despite the formation of biomolecular corona [33]. Although the understanding of the PC on nanoparticles has been advancing in recent years, little is known regarding whether PC formation could influence biomimetic nanoparticles’ cellular uptake, BBB penetration and targeting capacity for precision brain drug delivery.

**Fig. 1:**
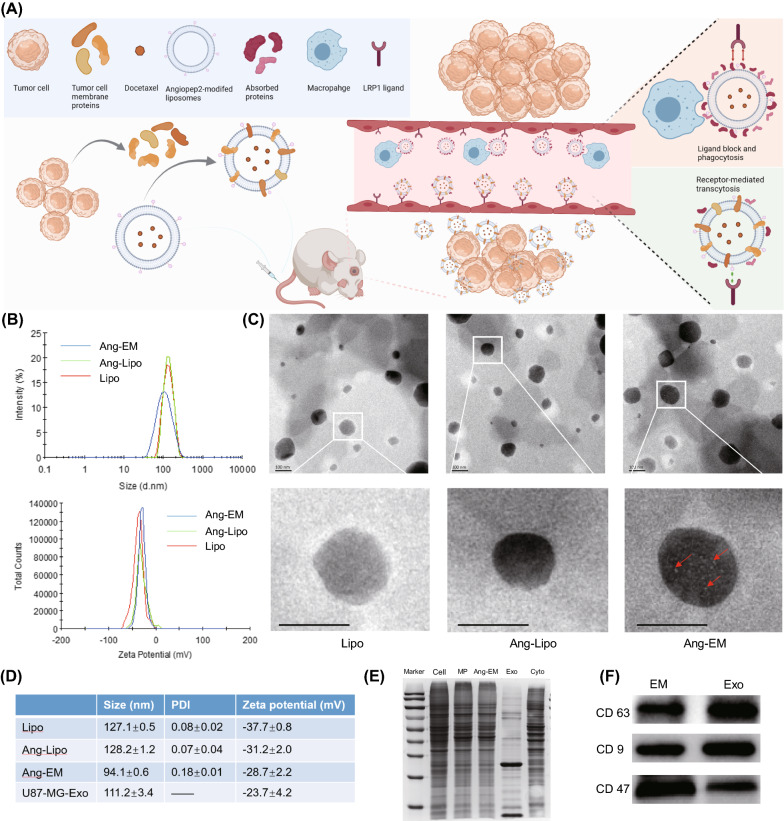
Study design and characterization of Ang-EM. **A** Illustration of DTX@Ang-EM preparation, avoiding protein corona formation, escaping phagocytosis, BBB penetration and GBM targeting. **B** Size distribution and zeta potential of Lipo, Ang-Lipo and Ang-EM. **C** TEM images of Lipo, Ang-Lipo and Ang-EM. Scale bar = 100 nm. **D** Summary and comparison of mean size, polymey distribution index and zeta potential of Lipo, Ang-Lipo and Ang-EM. **E** SDS-page analysis of protein profiles of Ang-EM. MP, membrane proteins; Exo, Exosomes; cyto, cytosolic proteins. **F** Western blot of protein markers of CD63, CD9 and CD47 on EM and Exo

In this study, we assume that chimeric proteins on biomimetic nanoparticle would occupy the surface and decrease the impact of biomolecular corona thus retaining its natural properties and targeting ability of ligand. Here, we developed EM by incorporating membrane proteins onto liposomes for brain tumor drug delivery. Ang was decorated onto EM for enhancing the BBB penetration ability. We found that Ang-EM have reduced PC formation by absorbing less serum proteins. Ang-EM showed superior GBM targeting ability and their mediated brain delivery of docetaxel (DTX) supressed significantly the GBM growth in mice (Fig. 1A). Findings from this study supported that this biomimetic strategy may be used for advanced drug delivery by retaining inherent stealth properties of nanocarriers.

## Materials and methods

### Cell culture and animals

bEnd.3 Brain-derived Endothelial cells, U87-MG/U87-MG-luc human glioblastoma cells and RAW264.7 macrophages were maintained in Dulbecco’s modified Eagle’s minimum essential medium supplemented with 10% fetal bovine serum (FBS) (VivoCell, Shanghai, China). Cells were incubated at 37 °C in humidified air with 5% CO_2_. Animals were obtained from SJA Laboratory Animal Co., Ltd (Hunan, China). Animal studies were approved by the Department of Laboratory Animals of Central South University.

### Preparation, characterization and drug loading of nanoformulations

U87-MG cells-derived exosomes were prepared as described previously [34]. Briefly, the supernatant of U87-MG cells culture was collected (48 h after incubation), and differentially centrifuged and filtered through a 0.2 μm filter followed by ultracentrifugation at 110,000 × g for 70 min and washed with phosphate-buffered saline (PBS). Cell membrane proteins of U87-MG cells were extracted using a Mem-PER™ Plus Membrane Protein Extraction Kit (Thermo Fisher Scientific, USA) according to manufactures’ instructions. Liposomes (Lipo) were synthesized by the thin layer evaporation (TLE) followed by the extrusion method. Briefly, 10 mg of DMPC, 2 mg of DSPE-PEG_2000_ and 1 mg of cholesterol were dissolved in methanol (9 mL). The solvent was evaporated using a rotatory evaporator (N-1300, EYELA, Japan) to form a thin film. Films were hydrated with water for 30 min. Lipid suspension was extruded through 200-nm cellulose acetate membranes (Whatman, USA) for 15 times using Avanti Mini-Extruder (Avanti Polar Lipids, USA). For the construction of EM, lipid films were hydrated with 100 μL of PBS containing 70 μg of membrane proteins. Ang (TFFYGGSRGKRNNFKTEEY) was obtained from Ruixi Biological Technology Co., Ltd. (Xi’an, China). Ang-conjugated Lipo or EM were developed by introducing DSPE-PEG_2000_-Angiopep-2 to the lipids. Ang on DSPE-PEG_2000_ was characterized by ^1^H-NMR.

Size distribution, polydispersed index (PDI) and zeta potentials of Lipo, Ang-Lipo, Ang-EM and U87-MG cells-derived exosomes were analyzed using Zetasizer (ZS90, Malvern, UK). Also, nanoparticle tracking analysis (NTA) was performed to assess the size distribution of U87-MG cells-derived exosomes (Nanosight NS300, Malvern, UK). Morphology was observed by transmission electron microscopy (TEM). Images were captured using a Tecnai G2 Spirit TWIN Electron Microscope (FEI, USA). Protein profiles of Ang-EM and exosomes were compared by Coomassie brilliant blue staining (Beyotime, China). The presence of protein markers CD47 (ab175388, Abcam, UK), CD9 (ab92726, Abcam, UK) and CD63 (ab216130, Abcam, UK) were detected via western blotting and analyzed using a gel imaging system (ChemiDoc™ Touch, Bio-Rad, USA).

Drug-loaded formulations were obtained by add 1 mg of docetaxel (DTX) to the lipids (13 mg) before thin layer formation. Free drugs were removed by ultrafiltration (100 kDa). The size distributions, zeta potentials, drug-loading capacity and encapsulation efficiency of nanoparticles after loading DTX were measured. The amount of DTX loaded into nanoparticles were measured by HPLC (HA-20 T, Shimadzu, Japan). The release profile of DTX-loaded nanoparticles under shaking (100 rpm) was evaluated using an ultrafiltration tube with 10 kDa cutoff (Millipore) against PBS.

### Cellular uptake and in vitro BBB transport

Lipo, Ang-Lipo and Ang-EM were labeled with fluorescent dye DiL (Yeason, China). Briefly, 4 μL of DiL (1 mg/mL) was added to the lipids dissolved in methanol as described above. Unbounded DiL was removed by using a 10 kDa ultrafiltration tube. bEnd.3 cells and U87-MG cells in 24-well plate were treated with DiL-labeled nanoparticles for 3 h followed by fixing with paraformaldehyde and nuclei staining with DAPI (Beyotime, China). Cellular internalization was observed using Olympus IX73 fluorescence microscope (Olympus, Japan).

The in vitro BBB model was developed as previously described [9]. Briefly, cell culture inserts (Corning, NY, USA), pre-coated with human plasma fibronectin (50 μg/mL) for 1 h, were put into 24-well plates. Then, 1 × 10^4^ bEnd.3 cells were seeded on each upper chamber insert and cultured with 0.5 ml of medium. After the cell confluency in the upper chamber reached 80%, the bottom chamber was seeded with 1 × 10^4^ U87-MG cells filled with 1.5 ml of culture medium. To evaluate the transcytosis, DiL-labeled nanoparticles were added to the upper chamber of the in vitro BBB model. Cells in the lower chamber were imaged by fluorescence microscope at different time points to observe the uptake of nanoparticles.

### Cytotoxicity and cell cycle assay

To assess the cytotoxicity of DTX@Ang-EM, U87-MG cells were seeded into 96-well plates at a density of 5 × 10^3^ cells per well overnight and then treated with free DTX or DTX nanoformulation for 24 h. Cell viability was assessed by CCK8 assay (NCM biotech, China) by measuring the absorbance at 450 nm using an Infinite F50 microplate reader (Tecan, Switzerland). For cell cycle assay, U87-MG cells were seeded into 6-well at a density of 1 × 10^4^ per well and treated with free DTX or DTX nanoformulation at an equivalent DTX dose (5 μg/mL) for 24 h. Cell cycle assay kit (Beyotime, China) was used according to the manufacturer’s instructions. Cell cycles after treatment were analyzed using flow cytometry (BD Biosciences, USA).

### 3D tumor spheroid

U87-MG cells were cultured and embedded in Matrigel (BD, USA) to form spheroids. Live spheroids were imaged. The tumor spheroids were incubated with different drug-loaded formulations for 48 h. Images of spheroids were imaged to observe size. Also, tumor spheroids were incubated with DiL-labeled nanoparticles for four hours and washed with PBS. Nuclei were stained using Hoechst 33258. Z-stack scanning images were taken using confocal microscopy (Leica TCS SP8 X, Leica, Germany) to observe the penetration of nanoparticles into intact and live spheroids. Spheroids were incubated with drug-loaded nanoparticles at equivalent DTX concentration (10 μg/mL) for two days and imaged under microscope to observe the in vitro antitumor effects.

### Protein corona formation and analysis

To form protein corona in vitro, nanoparticles were incubated directly with FBS at 37℃ for 1h [35]. Nanoparticles were separated from excess proteins by centrifugation (15000 g × 15 min) followed by washing with PBS for twice at the same centrifugation condition. Size distribution, PDI and zeta potentials for nanoparticles before and after protein corona formation were compared. TEM was performed to observe morphology of protein corona on nanoparticles. BCA assay and SDS-page analysis of profiles of protein corona on nanoparticles were also performed.

Proteins were characterized by mass spectrometry (MS). Briefly, protein samples were prepared by SDT lysis as previously described [36]. Samples were analysed on a nanoElute (Bruker, Bremen, Germany) coupled to a timsTOF Pro (Bruker, Bremen, Germany) equipped with a CaptiveSpray source. Peptides were separated on a 25 cm × 75 μm analytical column, 1.6 μm C18 beads with a packed emitter tip (IonOpticks, Australia). The column temperature was maintained at 50 °C using an integrated column oven (Sonation GmbH, Germany). The column was equilibrated using 4 column volumes before loading sample in 100% buffer A (99.9% MilliQ water, 0.1% FA) (Both steps performed at 800 bar). Samples were separated at 300 nl/min using a linear gradient as follows: 3% buffer B for 3 min, 3–28% buffer B for 70 min, 28–38% buffer B for 7 min,38–100% buffer B for 5 min, hold in 100% buffer B for 5 min.

The timsTOF Pro (Bruker, Bremen, Germany) was operated in PASEF mode. Mass Range 100 to 1700 m/z, 1/K0 Start 0.6 V⋅s/cm2 End 1.6 V⋅s/cm2, Ramp time 100 ms, Lock Duty Cycle to 100%, Capillary Voltage 1500 V, Dry Gas 3 l/min, Dry Temp 180 °C, PASEF settings: 10 MS/MS scans (total cycle time 1.16 s), charge range 0–5, active exclusion for 0.4 min, Scheduling Target intensity 20,000, Intensity threshold 2500, CID collision energy was 42 eV. The MS data were analysed by label-free quantification using MaxQuant software version 1.6.14.0. Proteins were identified by data-dependent acquisition based on the Uniprot_Bovine_46766_20210308 peptides database.

To evaluate the effects of protein corona formation on cellular uptake of nanoparticles, nanoparticles were labeled by DiL and incubated with FBS to form PC and then incubated with U87-MG cells and Raw264.7 cells to visualize the cellular uptake.

### Biodistribution

In situ U87-MG GBM model was developed as previously described with modification [37]. Mice were intracranially implanted with 2 × 10^6^ U87-MG cells using a 10 μl microsyringe pump. Tumor-bearing mice were administrated with DiR-labeled nanoparticles or free DiR via *i.v.* injection. 4, 8 and 24 h post-injection, fluorescence was measured using IVIS spectrum (PerkinElmer, USA). Ex vivo biodistribution in brains and other major organs was also inspected.

### In vivo antitumor study

Mice with GBM were treated with PBS, DTX, DTX@Lipo, DTX@ANG-Lipo or DTX@ANG-EM (5 mg/kg DTX) four times with an interval of three days. Bioluminescence images were obtained by IVIS Spectrum (PerkinElmer, USA). After intervention, mice were sacrificed, blood samples and tumor and major organs were excised and weight. Major organs were fixed in 4% PFA and stained with H&E. Tumors were stained by TUNEL to observe cell death. Plasma levels of ALT, AST, BUN, Cr were measured using assay kits (Rayto, China). To observe the tumor penetration of nanoparticles, GBM-bearing mice were given Dio-labeled Lipo, Ang-Lipo or Ang-EM for one injection, brain tissues were collected, fixed, sliced and stained with DAPI and observed under fluorescence microscope.

### Statistical analysis

Data were presented as the mean ± SD. A two-tailed Student’s t-test was applied to test the statistical significance of the difference between two groups, one-way analysis of variance (ANOVA) was applied to test the statistical significance of difference among three or more groups. The statistical significance was set at * *P* < 0.05, ** *P* < 0.01 and ***P < 0.001.

## Results and discussion

### Preparation and characterization of DTX@Ang-EM

EM was prepared by integrating membrane proteins of U87-MG cells into liposomes to mimic the size and protein composition of exosomes. Ang was used to enhance the GBM-targeting effects and Ang-conjugated to DSPE-PEG was evidenced by ^1^H-NMR (Additional file 1: Figure S1). Dynamic light scattering (DLS) demonstrated a slightly larger mean size of Ang-Lipo than blank lipo, but a smaller mean size was observed for Ang-EM (Fig. 1B, D). The size distribution of Ang-EM is similar to exosomes derived from U87-MG cells as detected by NTA (Additional file 1: Figure S2). Zeta potentials of blank Lipo, Ang-Lipo and Ang-EM were − 37.7 ± 0.8, − 31.2 ± 2.0 and − 28.7 ± 2.2 mV, respectively, indicating that Ang modification decreased slightly the zeta potential for all nanoparticles. TEM images (Fig. 1C) showed similar results for particle size as measured by DLS. Ang modification have little influence in the morphology of Lipo, while protein particles could be observed on the surface of EM (Fig. 1C). SDS-page analysis showed that EM express most membrane proteins of U87-MG cells (Fig. 1E). Also, western blot showed comparable levels of protein markers including CD47, CD9 and CD63 in EM and exosomes (Fig. 1F). Those results demonstrated that EM could mimic exosomes closely in regards to size and protein markers. Previous reports also produced exosome-like vesicles by integrating membrane proteins to liposomes [38].

A slightly increased size of nanoparticles was observed after DTX loading (Fig. 1D, Additional file 1: Table S1), and similar phenomenon has been reported previously [7, 39]. There was no significant difference for the entrapment efficiency and the drug loading capacity of DTX loaded into equivalent lipid levels of nanoparticles (Additional file 1: Table S1). Drug release profiles of DTX from nanoparticles were similar, while the 72 h cumulative release of DTX@Ang-EM was lower than DTX@Lipo and DTX@Ang-Lipo (Additional file 1: Figure S3).

The stability test showed that nanoparticles were stable under storage with no significant change in size and PDI. Although the zeta potentials were decreasing during 14 days of storage (Additional file 1: Figure S4), suggesting potentially decreased colloidal stability with storage durations.

### Cellular uptake and BBB transport

**Fig. 2:**
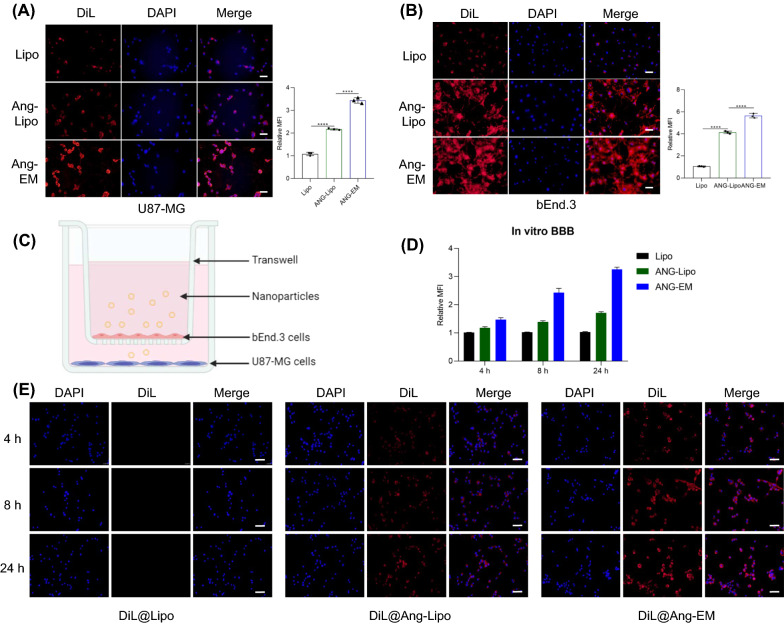
Cellular uptake and in vitro BBB penetration study. **A** Cellular uptake and analysis of mean fluorescence intensity of nanoparticles by U87-MG cells. Scale bar = 100 μm. **B** Cellular uptake and analysis of mean fluorescence intensity of nanoparticles by bEnd.3 cells. Scale bar = 100 μm. **C** BBB model by transwell, bEnd.3 cells were seeded onto the upper chamber coated with human plasma fibronectin, U87-MG cells were seeded onto the lower chamber, nanoparticles were added to the media of upper chamber. **D** Analysis of fluorescence intensity of nanoparticles in the U87-MG cells seeded on to the lower chamber at different time points. **E** Fluorescence images of cellular uptake of nanoparticles by the U87-MG cells seeded on to the lower chamber at different time points. Scale bar = 100 μm. *** *P* < 0.001

We firstly tested the cellular uptake of nanoparticles by U87-MG cells and by bEnd.3 cells Ang-EM showed the most significant cellular uptake for both U87-MG cells and bEnd.3 cells (Fig. 2A, B). Then, the in vitro BBB model was developed to test the transcytosis and BBB penetration ability of nanoparticles (Fig. 2C). As a result, Lipo showed minimal penetration as no significant fluorescence was observed in U87-MG cells in the lower chamber. Ang modification enhanced the penetration of Lipo while Ang-EM showed the most significant cellular uptake in the lower chamber (Fig. 2D). More importantly, a time-dependent cellular uptake was observed for Ang-Lipo and Ang-EM (Fig. 2E), demonstrating the enhanced BBB penetration ability of EM with Ang modification.

### Tumor penetration and biodistribution study

**Fig. 3:**
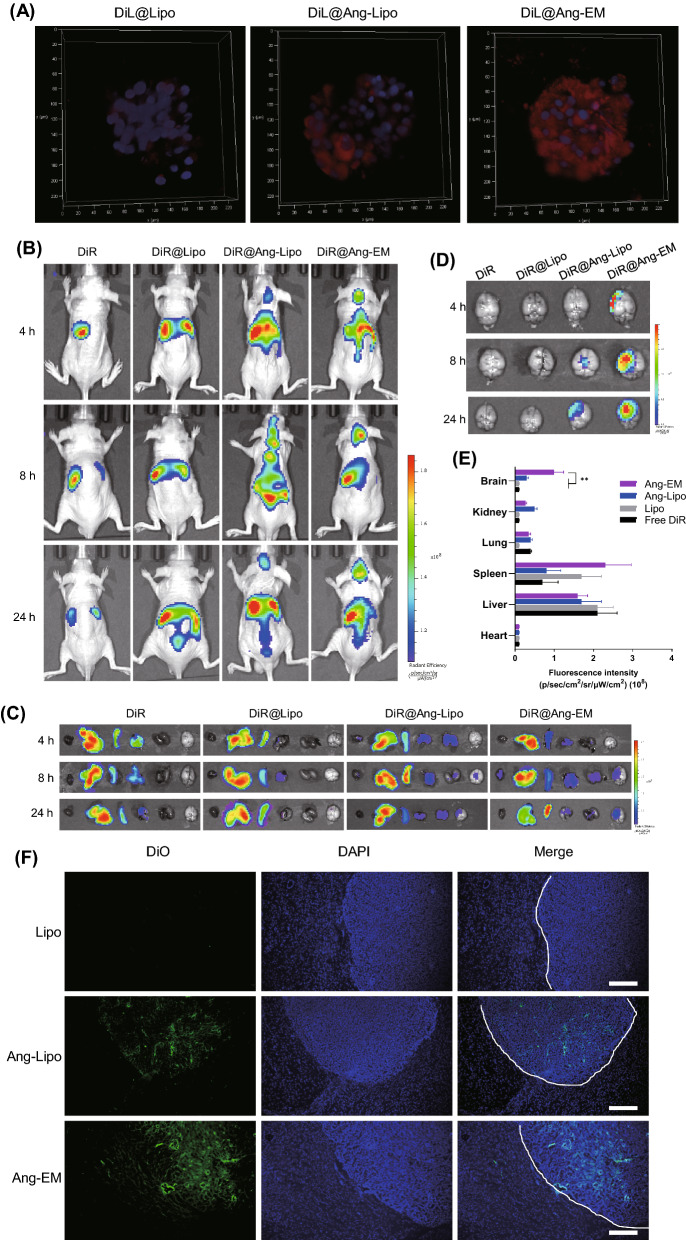
Tumor penetration and biodistribution study. **A** Z-stack scanning showing penetration of nanoparticles (red) in to 3D tumor spheroid. **B** In vivo biodistribution of nanoparticles in mice with GBM. (n = 3). **C** Ex vivo biodistribution of nanoparticles in mice with GBM. **D** Brain distribution of nanoparticles showing brain tumor targeting effects. **E** Analysis of fluorescence intensity of nanoparticles in major organs. **F** Distribution of nanoparticles in the brain of GBM-bearing mice after single injection. Brain tumor boundary was highlighted by white dotted line. ** P < 0.01

3D tumor spheroid of U87-MG cells was developed to evaluate the penetration ability of nanoparticles. Blank Lipo showed little ability to penetrate the spheroid, Ang-Lipo showed enhanced penetration to the spheroid while Ang-EM showed the strongest penetration to the spheroid (Fig. 3A).

The brain-tumor targeting ability and biodistribution of nanoparticles were evaluated and compared. Mice with in situ GBM were administrated with DiR-labelled nanoparticles or free DiR through tail vein injection. Fluorescence images were obtained using IVIS at 4, 8 and 24 h post-injection (Fig. 3B). DiR-labelled Ang-Lipo and Ang-EM reached and accumulated at the tumor site after injection, but free DiR and DiR@Lipo showed no fluorescence signals in the brain (Fig. 3C, D), indicating that Ang modification effectively enhanced the brain targeting ability of nanoparticles. Analysis of fluorescence intensity revealed significant higher accumulation of Ang-EM at the brain tumor than Ang-Lipo and other controls 24 h after injection (Fig. 3E). Fluorescence scanning microscope demonstrated significant fluorescence intensity of Ang-EM at the tumor site (Fig. 3F). More importantly, Ang-EM distributed mainly within the tumor boundary, suggesting superior tumor targeting ability of Ang-EM (Fig. 3F). The CD47 may have played a role in the improved performance of brain delivery of Ang-EM as compared to Ang-Lipo. The CD47 “don't eat me” signal is of importance for the mechanism of immune evasion of malignant cells by inhibiting phagocytosis [40], which may have facilitated the accumulation of Ang-EM at the brain tumor site. For the biodistribution of Ang-EM, strong fluorescence was also detected in the body of GBM-bearing mice. Similar finding has also been reported [41]. The LRP-1 is a widely expressed signaling receptor [42]. Although the Ang modification improved the brain delivery, it may also lead the nanoparticle to other sites, causing off-target effects. However, this phenomenon may also provide this strategy with other therapeutic opportunities at other tissues.

### Protein corona formation

**Fig. 4:**
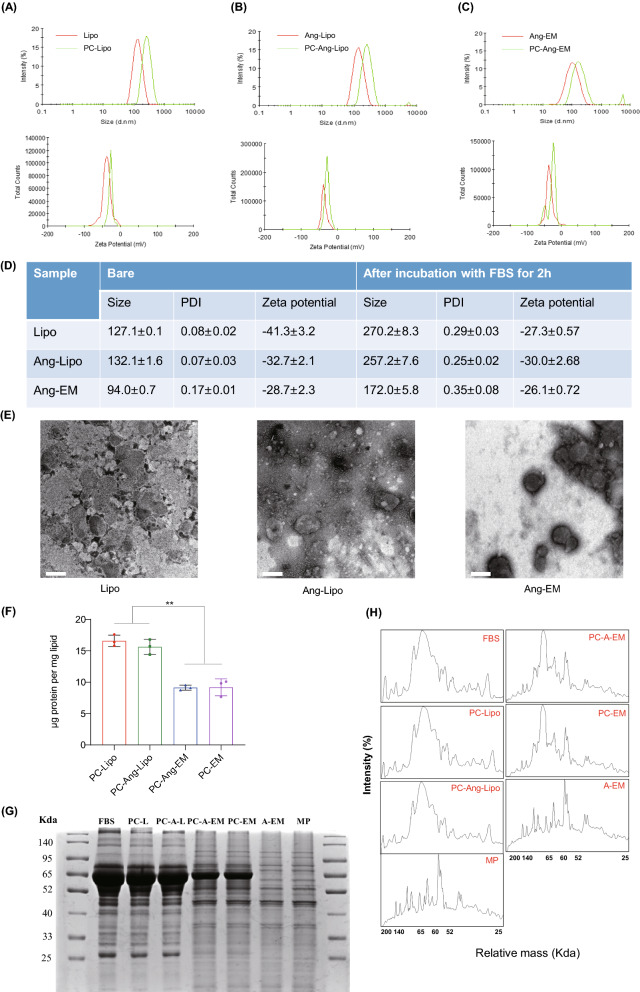
Formation of protein corona (PC) of nanoparticles. **A** Size distribution and zeta potential of Lipo and PC-Lipo. **B** Size distribution and zeta potential of Ang-Lipo and PC-Ang-Lipo. **C** Size distribution and zeta potential of Ang-EM and PC-Ang-EM. **D** Summary and comparison of mean size, PDI and zeta potential of nanoparticles before and after PC formation. **E** TEM images of nanoparticles after PC formation. Scale bar = 200 nm. **F** Rations of protein/lipid for nanoparticles after PC formation. **G** SDS-page analysis of protein profiles of nanoparticles after PC formation. FBS, fetal bovine serum; PC-L, Liposomes with protein corona; PC-A-L, Ang-Lipo with protein corona; PC-A-EM, Ang-EM with protein corona; A-EM, Ang-EM; MP, membrane proteins of U87-MG cells. **H** Intensity of bands relative to proteins onto surface of nanoparticles (analyzed by ImageJ, x-axis represents molecular weight while y-axis represents the intensity)

Bare nanoparticles and PC-formed nanoparticles were characterized by physicochemical properties and morphology. Bare nanoparticles were homogeneous in size with relatively lower PDI than PC-formed nanoparticles; however, after PC formation, all nanoparticles had increased diameters (nearly doubled) and PDI, but decreased zeta potentials (Fig. 4A–C). As observed in TEM images (Fig. 4E), PC-coated nanoparticles were not homogeneous in size as bare nanoparticles and the size distribution of PC-coated nanoparticle is in agreement with DLS result. Besides, Proteins distributed more significantly on Lipo, Ang-Lipo than Ang-EM (Fig. 4E). We next investigated the protein profiles of PC coated on nanoparticles. Firstly, the amount of proteins coated on nanoparticles was quantified for equal quantity of lipids by BCA assay. As a result, PC-Lipo and PC-Ang-Lipo showed similar levels of protein (about 15 μg proteins/1 mg lipids) coated on nanoparticles, PC-Ang-EM and PC-AM showed similar but significantly lower protein levels (about 8 μg proteins/1 mg lipids) than Lipo and Ang-Lipo (Fig. 4F). Then, proteins on equal quantities of lipids were visualized by gel electrophoresis (SDS-PAGE) and Coomassie Brilliant Blue staining. PC-Ang-EM showed fewer protein bands (Fig. 4G) and weaker intensity (Fig. 4H) compared to PC-Lipo and PC-Ang-Lipo. PC-Ang-EM and PC-EM showed similar protein corona compositions. Collectively, these results demonstrated that EM absorbed fewer proteins than Lipo and Ang modification has little influence in the protein corona formation.

**Fig. 5:**
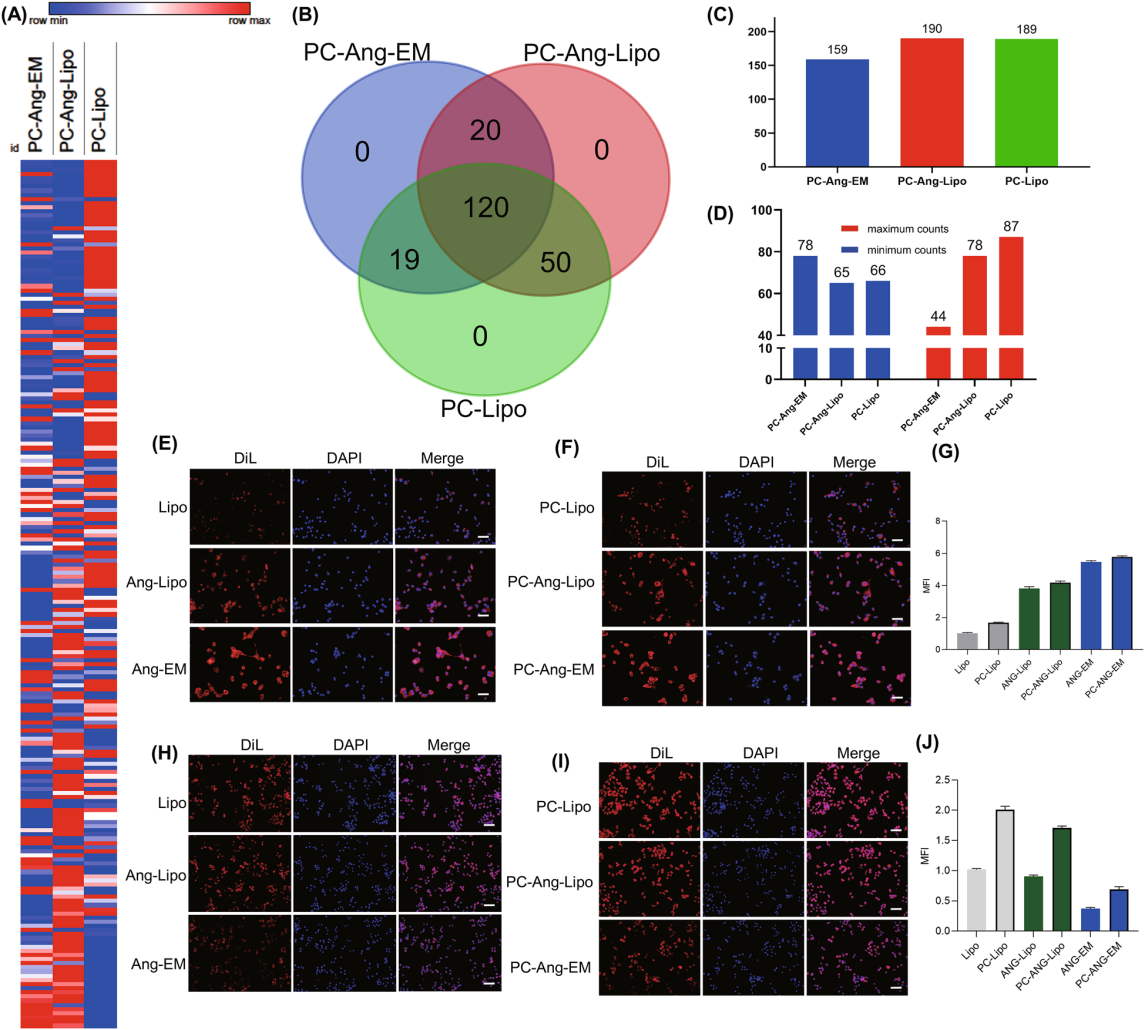
Proteome analysis and comparison of cellular uptake of nanoparticles after protein corona formation. **A** Heatmap of protein composition of PC-formed nanoparticles. **B** Venn diagram showing common and exclusive proteins on nanoparticles. **C** Comparison of number of proteins identified. **D** Comparison of maximum and minimum protein identified on different nanoparticles. **E** Cellular uptake of bare nanoparticles by U87-MG cells. **F** Cellular uptake of PC-formed nanoparticles by U87-MG cells. **G** Comparison of mean fluorescence intensity for nanoparticles uptaken by U87-MG cells. **H** Cellular uptake of bare nanoparticles by Raw264.7 macrophages. **I** Cellular uptake of PC-formed nanoparticles by Raw264.7 macrophages. **J** Comparison of mean fluorescence intensity for nanoparticles taken up by Raw264.7 macrophages. All scale bar = 100 μm

For proteome analysis, we generated Venn diagram and heatmap using Versatile matrix visualization, the intensity of each protein was normalized by Z-score for the heatmap. As a result, a total of 209 proteins were identified. Ang-EM, Ang-Lipo and blank Lipo were significantly different in PC composition from the heatmap (Fig. 5A); however, there is no exclusive protein identified among all nanoparticles (Fig. 5B). PC-Ang-EM showed less proteins while PC-Ang-Lipo and PC-Lipo showed similar numbers of proteins (Fig. 5C). Besides, we compared the counts of maximum and minimum proteins on each PC-formed nanoparticles and PC-Ang-EM showed more minimum counts but less maximum counts (Fig. 5D). These combined results demonstrated that Ang-EM was influenced not as seriously as Ang-Lipo and blank Lipo by the PC formation. The numbers of proteins in the corona could be affected by various factors and previous studies identified different numbers of proteins coated onto nanoparticles [43–45]. The incubation time have impact on the proteins identified on the PC and longer incubation time may increase the number and enrichment of proteins [35, 44, 45]. The separation method also affects the results of protein corona analysis. A recent study identified similar number of proteins to our study by the centrifugation method, despite the affinity chromatography method used in that study showed higher collecting efficiency than centrifugation method, the number of proteins was still far less than the total proteins that can be identified in plasma [44].

For PC-formed nanoparticles, we tested the cellular uptake by U87-MG cells and phagocytosis by macrophages. It can be observed that Lipo, Ang-Lipo and Ang-EM were uptake by U87-MG cells from low to high (Fig. 5E), PC-formed nanoparticles showed enhanced cellular uptake (Fig. 5F), demonstrating that PC formation could facilitate the cellular uptake process (Fig. 5G). For macrophage phagocytosis, Ang-EM showed the lowest cellular uptake for the membrane proteins such as CD47 from tumor cell, while Lipo and Ang-Lipo showed similar macrophage uptake (Fig. 5H). PC-nanoparticles showed significantly enhanced macrophage uptake (Fig. 5I) while the trend keeps consistent with bare nanoparticles (Fig. 5J), demonstrating that PC formation promoted the immune clearance. It has been reported that proteins absorbed to nanoparticles can bind to their receptors on macrophages thus promoting phagocytosis [30]. But the immune escape ability of membrane proteins of tumor cells was not masked by PC as PC-formed Ang-EM showed still the lowest macrophage phagocytosis (Fig. 5J).

### Antitumor effect of DTX@Ang-EM

**Fig. 6:**
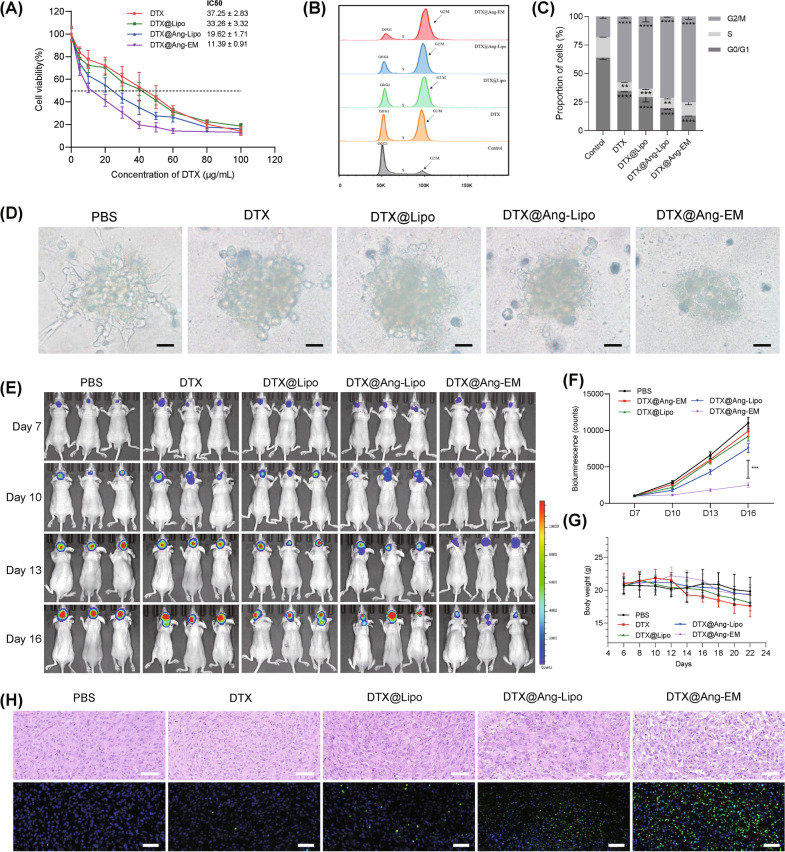
Antitumor efficacy of DTX@Ang-EM and control nanoparticles. **A** Relative cell viability of U87-MG cells after incubation of different concentration of DTX formulations. **B** Cell cycle assay by flow cytometry. **C** Quantitative analysis of cell cycle showing inhibition effects of different formulations. **D** 3D tumor spheroid after incubation with different formulations. Scale bar = 50 μm. **E** Bioluminescence of U87-luc cells showing the GBM growth in mice before and after treatment. **F** Quantitative analysis of bioluminescence intensity showing GBM growth. **G** Body weight of GBM-bearing mice before and after treatment. **H** H&E staining and TUNEL analysis of GBM tissues after treatment. Scale bar = 100 μm. ** P < 0.01, *** P < 0.001, **** P < 0.0001

The in vitro antitumor effect against U87-MG cells of free DTX, DTX@Lipo, DTX@Ang-Lipo and DTX@Ang-EM were evaluated and compared. A dose–response relationship of cytotoxicity was observed (Fig. 6A). The calculated IC50 for free DTX, DTX@Lipo, DTX@Ang-Lipo and DTX@Ang-EM were 37.25, 33.26, 19.62 and 11.39 μg/mL, respectively. Liposomes-mediated delivery enhanced slightly the cytotoxicity of DTX, while Ang modification further increased the cytotoxicity. The enhanced cellular uptake and the increased concentration of drugs in tumor cells (Fig. 2) may have contributed to the enhanced cytotoxicity. Cell cycle assay was performed to assess the cytotoxic effects of DTX@Ang-EM on U87-MG cells (Fig. 6B). As a result, the proportion of U87-MG cells in G2/M phase was significantly increased, while the proportion in G0/G1 phase was significantly decreased after incubation with free drug or nanoformulations. More importantly, DTX@Ang-EM showed the most significant inhibition effects on cell cycle (Fig. 6C). Further, we developed 3D spheroids of U87-MG cells to evaluate the cytotoxicity of DTX@Ang-EM. Similar to results in 2D cell culture, DTX@Ang-EM showed the most significant disruption of 3D spheroids than other groups (Fig. 6D). Free DTX, DTX@Lipo decreased the size of tumor spheroids, but failed to disrupt the spheroid.

The in vivo antitumor effects of DTX@Ang-EM was evaluated in mice with in situ GBM. The bioluminescence shows the GBM mass (Fig. 6E). Mice were treated with PBS, DTX, DTX@Lipo, DTX@Ang-Lipo or DTX@Ang-EM (5 mg/kg DTX) four times via *i.v.* injection, with an interval of three days. Free DTX exhibited limited suppression of tumor growth in GBM-bearing mice. DTX@Lipo and DTX@Ang-Lipo showed enhanced antitumor efficacy compared to free DTX, while DTX@Ang-EM showed the most significant suppression of GBM growth (Fig. 6F). H&E staining and TUNEL analysis of in situ GBM mice models confirmed significant cell death in mice receiving DTX@Ang-EM (Fig. 6H).

The safety has been a major concern for the use of nanocarriers for drug delivery. We monitored the change of body weight of mice, and the body weight of GBM-bearing mice receiving free DTX treatment was decreasing during treatment while of nanoparticles-mediated DTX delivery alleviated the decrease of body weight (Fig. 6G), suggesting potential safety of nanoparticle-mediated DTX delivery. GBM-bearing mice receiving free DTX treatment exhibited significant increase of serum levels of alanine aminotransferase (ALT) and aspartate aminotransferase (AST), demonstrating damages to liver functions; however, DTX@Ang-EM and other nanoparticles showed no obvious increased of these indexes (Additional file 1: Figure S5). For blood urea nitrogen (BUN) and creatinine (Cr) levels, there was no significant difference among all groups. Besides, H&E staining of major organs showed no damage (Additional file 1: Figure S6). Those results demonstrated improved safety of Ang-EM-mediated DTX delivery for GBM therapy.

## Conclusions

In this study, we developed an EM by integrating cell membrane proteins into liposomes as a biomimetic drug delivery system for brain tumor drug delivery. PC formation has minor influence in properties of Ang-EM when exposed to the biological environment. EM escaped the phagocytosis by macrophages and protected the targeting ligand Ang and retained the BBB penetration ability. DTX@Ang-EM targeted the GBM and supressed tumor growth with high compatibility and improved safety. Ang-EM provided a promising delivery platform for targeted brain drug delivery and GBM therapy.

## Supplementary Information

**Additional file 1: Figure S1**. 1H-NMR study showing the existence of Ang conjugated to DSPE-PEG. **Figure S2**. Size distribution of exosomes derived from U87-MG cells by nanoparticle tracking analysis. **Figure S3**. DTX release profiles for different nanoformulations. **Figure S4**. Storage stability of different nanoparticles. **Figure S5**. Serum levels of ALT, AST, BUN and Cr in mice with GBM after treatment. **Figure S6**. H&E staining of major organs of mice with GBM after treatment. Scale bar = 200 μm. **Table S1**. Drug loading properties of nanoparticles.
