# Supplementary material for: Multifunctional exosome-mimetics for targeted anti-glioblastoma therapy by manipulating protein corona
Source: J Nanobiotechnology. 2021 Dec 6;19:405. doi: 10.1186/s12951-021-01153-3 (PMC8647369; doi:10.1186/s12951-021-01153-3)
Supplement: Supplementary file 1 — Additional file 1: Figure S1. 1H-NMR study showing the existence of Ang conjugated to DSPE-PEG. Figure S2. Size distribution of exosomes derived from U87-MG cells by nanoparticle tracking analysis. Figure S3. DTX release profiles for different nanoformulations. Figure S4. Storage stability of different nanoparticles. Figure S5. Serum levels of ALT, AST, BUN and Cr in mice with GBM after treatment. Figure S6. H&E staining of major organs of mice with GBM after treatment. Scale bar = 200 μm. Table S1. Drug loading properties of nanoparticles. [file 12951_2021_1153_MOESM1_ESM.docx]

**Supporting Information**

**Multifunctional exosome-mimetics for targeted anti-glioblastoma therapy by manipulating protein corona**

Jun-Yong Wu^1,2,3,#^, Yong-Jiang Li^1,2,3,#^, Jiemin Wang^4^, Xiong-Bin Hu^1,2,3^, Si Huang^1,2,3^, Shilin Luo^1,2,3^, Da-Xiong Xiang^1,2,3,*^

1 Department of Pharmacy, The Second Xiangya Hospital, Central South University, Changsha, China

2 Hunan Provincial Engineering Research Centre of Translational Medicine and Innovative Drug, Changsha, China

3 Institute of Clinical Pharmacy, Central South University, Changsha, China

4 Regenerative Medicine Institute (REMEDI), School of Medicine, College of Medicine, Nursing and Health Sciences, National University of Ireland Galway, Galway, Ireland

# Contribute equally to this work.

*Correspondence: Da-Xiong Xiang, Ph.D.

Department of Pharmacy, the Second Xiangya Hospital of Central South University, 139 Middle Renmin Road, Changsha 410011, China

Tel: +86-073185292129

Email: [xiangdaxiong@csu.edu.cn](mailto:xiangdaxiong@csu.edu.cn)


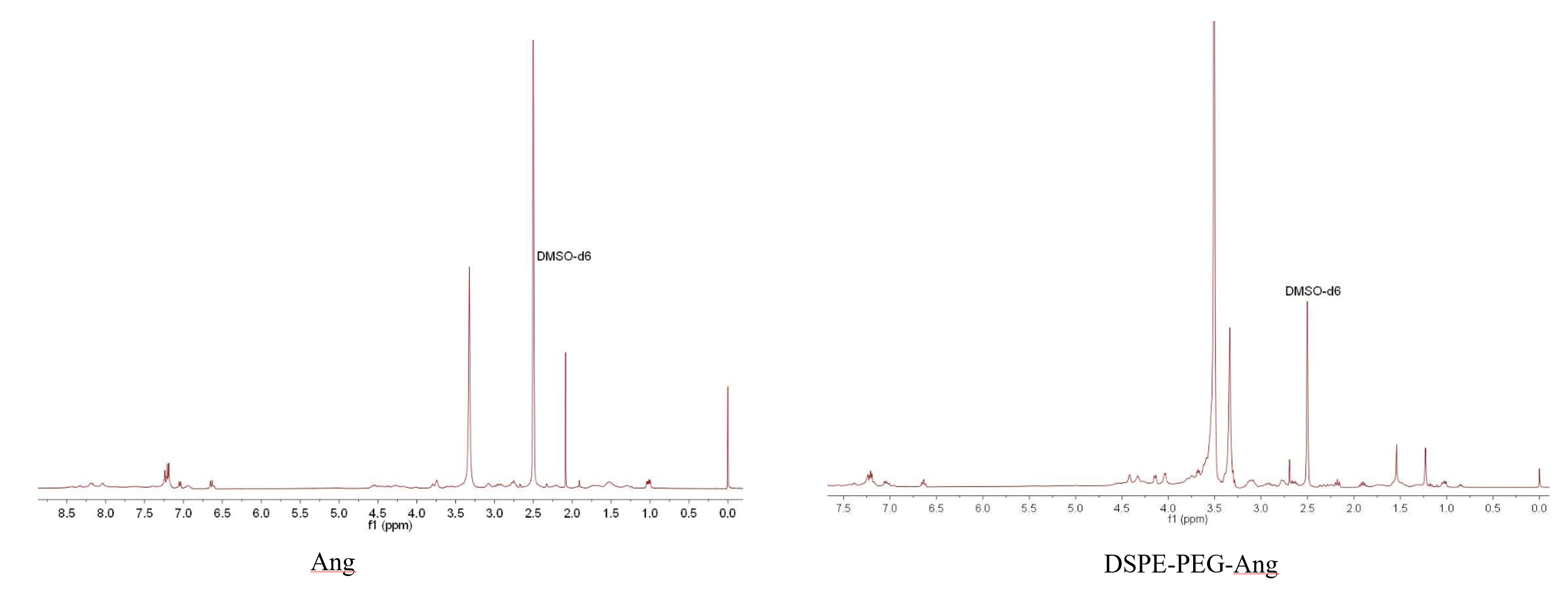


Figure S1. ^1^H-NMR study showing the existence of Ang conjugated to DSPE-PEG.

Figure S2. Size distribution of exosomes derived from U87-MG cells by nanoparticle tracking analysis.


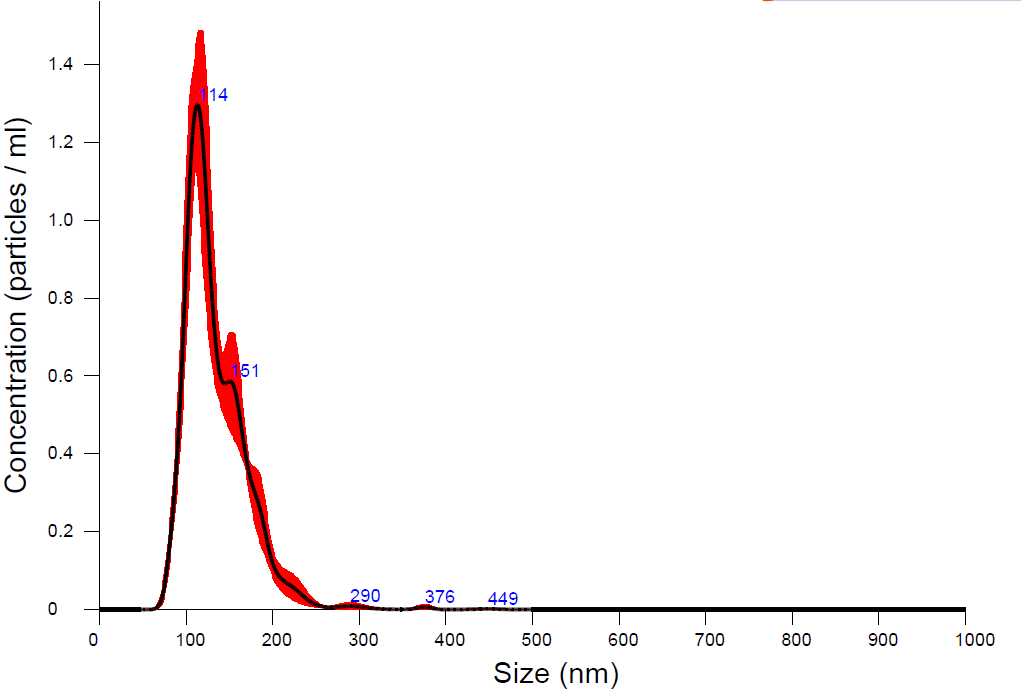


U87-MG-Exo

Table S1. Drug loading properties of nanoparticles.

| **Sample** | **Particle size** | **Zeta potential** | **DL (%)** | **EF (%)** |
| --- | --- | --- | --- | --- |
| DTX@Lipo | 128.60±0.98 | -41.20±1.20 | 5.16±0.87 | 72.5±12 |
| DTX@Ang-Lipo | 130.97±0.25 | -27.30±1.06 | 4.98±0.23 | 69.72±3.17 |
| DTX@Ang-EM | 95.41±1.04 | -25.43±0.95 | 4.8±0.43 | 67.55±6.03 |

DL, drug loading capacity; EF, encapsulation efficiency.

Figure S3. DTX release profiles for different nanoformulations.

Figure S4. Storage stability of different nanoparticles.

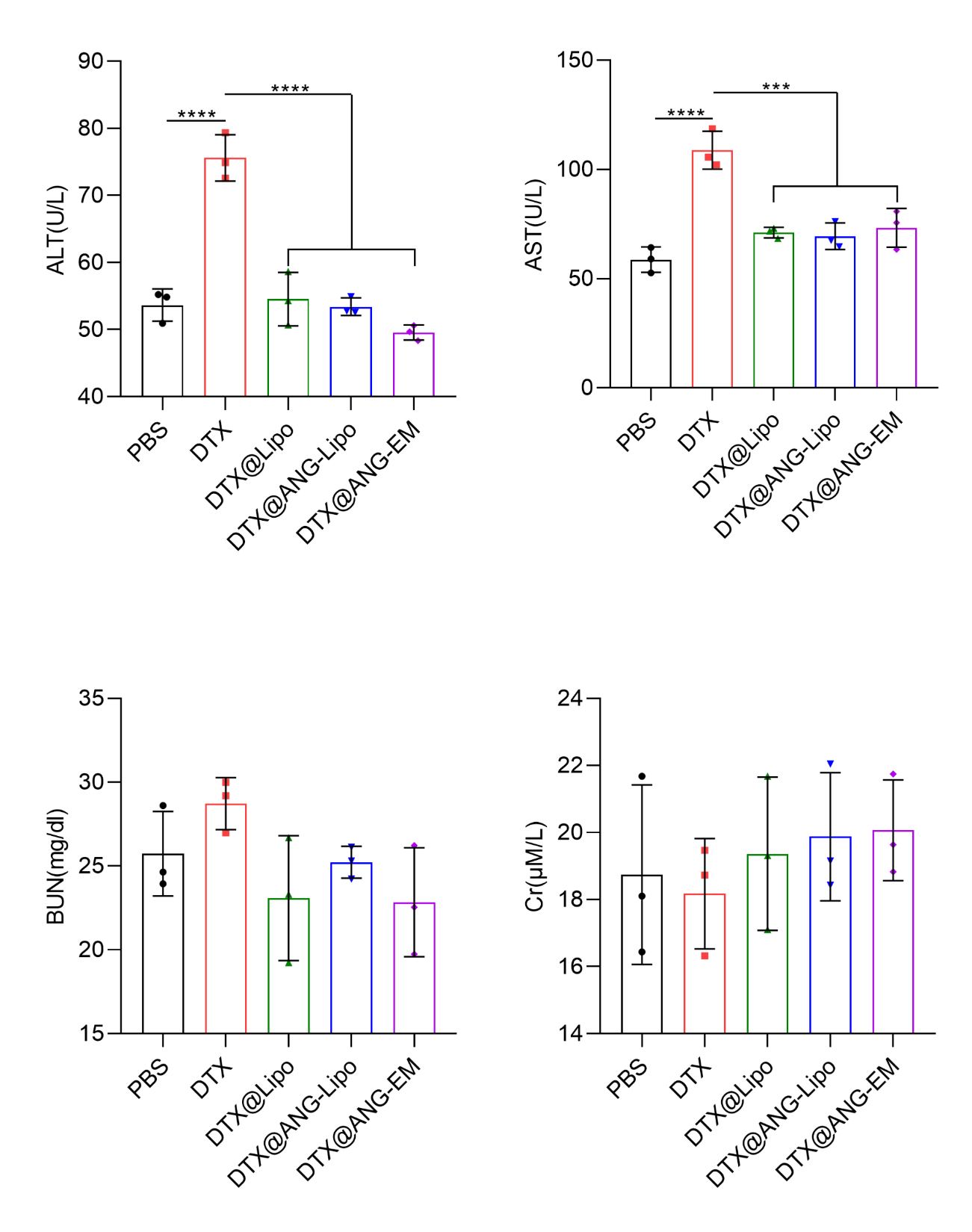


Figure S5. Serum levels of ALT, AST, BUN and Cr in mice with GBM after treatment.

Figure S6. H&E staining of major organs of mice with GBM after treatment. Scale bar = 200 μm.
